# Main Histological Types of Primary Epithelial Lung Tumours

**DOI:** 10.1038/bjc.1961.25

**Published:** 1961-06

**Authors:** Leiv Kreyberg

## Abstract

**Images:**


### MAIN HISTOLOGICAL TYPES OF PRIMARY EPITHELIAL LUNG TUMOURS

LEIV KREYBERG

*From the Institutt for Generell og Eksperimentell Patologi, Universitetet i Oslo, and WHO International Reference Centre for Lung Tumours*

Received for publication February 11, 1961

USUALLY lung cancer morbidity and mortality statistics refer to cases registered under No. 162 and 163 in the International List.

Already in 1937, in a discussion in the Medical Society in Oslo, I emphasized the very heterogenous tumour material embraced by the term " lung cancer " and stressed the necessity of distinguishing between the different histological types, when aetiological factors are being studied, as presumably the different histological types also represent biologically different tumour entities. In addition, always looms the uncertainty as to the tumour in question being a primary lung tumour or not.

Not until after the war came the opportunity to study this question systematically. At this time also the manifestation of a new lung cancer situation could be perceived, adding to the pertinency of such studies. The new situation is characterized by : a considerable and progressive increase in lung cancer cases especially in males in urban areas, and a much lower increase in males in rural areas and in women.

More than ten years ago I started collecting as many lung cancer cases as possible in Norway. A minor part were autopsy cases, the major part surgical, including considerable biopsy material.

From the very beginning of my effort to classify the pulmonary epithelial tumours according to histological types, I met methodological difficulties on one hand, and a good deal of scepticism from many colleagues on the other, both natural obstacles, intimately related.

In a series of papers (Kreyberg, 1937, 1954, 1956 and 1959) it has been claimed and shown that a separation of the histological types is not only fruitful, but essential in the study of " lung cancer ".

In order to conduct and to promote comparative studies of materials from different sources, it is very important that the different students use identical criteria and nomenclature.

It may therefore be useful to state in some detail the criteria and the nomenclature used by the author in recent years, essentially those laid down by a World Health Organization group of experts meeting in Oslo in 1958, consisting of Professors Dr. J. Delarue, Paris ; Dr. H. Hamperl, Bonn ; Dr. A. A. Liebow, New Haven ; Dr. R. W. Scarff, London ; Dr. E. Uehlinger, Zürich ; and Dr. L. Kreyberg, Oslo.

A few general remarks may be useful.

Most malignant tumours may occur in more or less differentiated forms, usually without this influencing the nomenclature, even if it may influence the clinician as to the best treatment and as to prognosis. A few examples may be mentioned.

A malignant melanoma may show a great variety of cells : spindle shaped, globoid or gigantic, and large areas may be without melanin. If, however, an area with melanin formation is identified, the diagnosis is : a malignant melanoma, with a possible addition, " little differentiated " or " highly polymorphous " or similar. No pathologist will diagnose " mixed melanoma and spindle cell sarcoma ", or " mixed melanoma and giant cell sarcoma ".

Another example. The nephroblastic embryonal tumours may in large areas appear with only spindle cells and possibly in a small area only show the characteristic glomerulus-Anlage pattern, giving the clue to the true diagnosis. The smallness of the special area does not invalidate the diagnosis, or make the specific diagnosis subsidiary to something else.

These examples should be kept in mind when we come to the typing of lung tumours, where many conventional pathologists tend to create mixed forms of different types, from the very findings of anaplastic or undifferentiated areas, or structures mimicing specific differentiations.

If we study a series of average primary so-called lung carcinomas we admittedly find a great variance in the size, the form and the differentiation of cells, even within one and the same tumour. Some cells are small, others may be middle sized, others again large. Some cells may be round, others polyhedral or columnar, others again more or less oval to spindle shaped and some may even be giant cells. Accordingly it is possible to apply a number of purely cyto-morphological designations : " small round cell ", " large round cell ", " fusiform ", " giganto-cellular "-carcinomas, etc.

If we come to cyto-functional differentiation, we may find some cells producing mucus, others not, and mucus of different kind, if finer methods are employed for identification. By some stains, as for instance mucicarmine, we actually identify a smaller number of mucus-producing cells than by the use of the alcian stains. A technical warning must be sounded. Sometimes necrotic nuclei are stained blue or green, and even certain degeneration products of the cytoplasm may take the stain and mimic mucus. Next, mucous cells of the bronchial walls may be infiltrated and embedded in the tumour tissue, more or less completely destroyed, and remnants of the mucus be taken as a product of the tumour cells proper. A strict analysis and a critical judgement is necessary, to avoid incorrect conclusions.

Another important cytological differentiation is keratinization with all stages up to complete keratosis with kerato-hyaline granules. Very often the keratinization is manifested only by a tendency of the cytoplasm to stain with eosine, orange-G, saffron and similar stains, which, however, are not specific. Many degenerating and necrotic cells may, on the other hand, take acid stains and resemble keratotic cells. A decision as to the true significance of the staining can sometimes be very difficult or impossible.

The various cyto-functional differentiations may be used in the choice of designation of the tumour, such as : " mucus producing carcinoma ", " keratinizing carcinoma ", etc.

If we next come to the architectural differentiation we may find : solid sheets, ribbons or garlands, buds and strings : we may find acinar or alveolar structures, even papillomas, but also completely anaplastic pictures. Again designations may be chosen from the histological pattern, such as : " carcinoma solidum ", " adenocarcinoma ", " carcinoma anaplasticum ", etc.

In the different systems of classification a great many combinations of the characteristics mentioned may be construed. In my own typing I have tentatively chosen the path of simplification. If anywhere in a tumour a definitely specific differentiation is found, however limited, this finding decides the type, undifferentiated areas being disregarded. If two different differentiations are found, the tumour is classified as " combined ".

The following types are used :

*Epidermoid carcinoma* is the designation for all tumours where any definite stratification or whorl formation or any, even traces of cornification have been found, due regard being paid to technical errors and to possible degenerative changes. The cytoplasmatic differentiation can be very marked or very slight. Very anaplastic tumours occur (Fig. 1).

*Small cell anaplastic carcinomas* may be composed of small polyhedral or of oval cells, usually with very scanty cytoplasm, in smears often with " naked " nuclei, " oat cells " (Fig. 2). The cells may appear in school, ribbons and garlands In many cases fragments may resemble the pictures found in " carcinoma *in situ* ", in the cervix (Fig. 3), and in others great likeness with very little differentiated squamous cell carcinomas (Fig. 4), the complete absence of keratinization then being the essential formal distinction.

In this group usually are also included small cell carcinomas with a growth of small polyhedral to slightly columnar cells, with formation of rosettes and even small lumina, sometimes containing a secretion resembling mucus (Fig. 5). It is possible that these tumours actually represent a special type, which ought to be separated from the other small cell carcinomas. This problem is worth a closer study.

From the age and sex occurrence and the finding of transitional forms it is probable that the small cell tumours actually are undifferentiated epidermoid carcinomas (Fig. 3 and 4).

*Adenocarcinoma* is the next large and important type. Here the basic morphologic criterium is gland-structures. If such structures are found in any part of the tumour, the base for diagnosis is established, even if large parts are anaplastic, large celled, giant celled or with the cells growing in solid heaps as a " carcinoma solidum " mimicking an epidermoid carcinoma (Fig. 6). Warning must be given that the epidermoid carcinomas, on the other hand, may mimic gland structures, when the cells of the periphery invade alveoles. Cytoplasmatic differentiation, in one or another part of the tumour, with mucus formation, or keratinization may guide to a safe conclusion as to type. A great variety of adenocarcinomas

---

EXPLANATION OF PLATES.

FIG. 1.—Epidermoid carcinoma with scattered keratinizing cells and no organized architecture.
FIG. 2.—" Oat cell " carcinoma with a uniform population of small oval cells.
FIG. 3.—Small cell anaplastic carcinoma with garland formation, no stratification, no trace of keratinization.
FIG. 4.—Small cell carcinoma ? with uncertain stratification and no keratinization—Undifferentiated epidermoid carcinoma ?
FIG. 5.—Small cell carcinoma with " rosette "—or " gland " like structures. No mucine formation.
FIG. 6.—Undifferentiated area in an adenocarcinoma (Fig. 7 and 8 ; Kreyberg, 1959).

1

2

3

Kreyberg.

4

5

6

are found, including the tumours by Nash and Stout (1958) designated " giant cell carcinomas of the lung ".

If these criteria described, are strictly observed, combined epidermoid adeno-carcinomas are found in human material, but rather rare—a few per cent of all, or less.

*The bronchiolo-alveolar carcinomas* have the well-known characteristic pattern. Certain difficulties may be encountered when being confronted with ovarian lung metastases and some plain primary papillary adenocarcinomas of the lung. This problem is, however, of minor quantitative importance.

*Mucous gland tumours*, mainly cylindromas, are limited to trachea and the larger bronchii, and these tumours are usually easily identified.

*The carcinoids* or *adenomas* are, likewise, in most cases easily recognized. Sometimes, however, when deeply infiltrating, a differential diagnosis versus the small cell anaplastic carcinomas must be remembered.

*Undifferentiated carcinomas* means tumours so undifferentiated that no clue is found permitting them to be included in any of the above types.

From the purely morphological point of view, how many of a total of un-selected lung cancers can now be identified according to the criteria mentioned ?

The best answer is not obtained from my own Norwegian material, because that was my first, and I did not keep a precise record of the number of cases discarded because of technical defects, and my own inability to come to a safe conclusion.

A good answer is, however, given by a Finnish material of 624 cases, studied and typed during April–July, 1960. The material, which will be reported upon later, was supplied by a series of Finnish laboratories through the co-operation of Professor Saxén. As " lung cancer " unspecified were recognized all cases where the histological material permitted a diagnosis of " carcinoma ". This was done in order to be in agreement with the clinicians and the statisticians. Some of the cases, could, however, not be used safely for typing, because of defects of the material, in quality or in quantity. A number of cases of good material, however, still left us in uncertainty as to type—undifferentiated carcinoma.

In this ordinary material of routine preparations some 10 per cent were discarded as unfit for typing, and of the remaining bteween 96 and 97 per cent could be typed and classified according to the criteria stipulated. Combined epidermoid-adenocarcinoma was diagnosed in 3 cases only.

I estimate that a similar situation has existed during my study of the Nor-wegian material.

During the some twelve years where the author has been occupied with attempts at a fruitful typing, the criteria have varied slightly, and even at the present stage it is evident that the final word has not been written on this subject. The classification and the criteria described in the present paper have, however, now reached a stage where the usefulness of the typing is evident, as will be shown in other papers to follow.

### SUMMARY

A group of experts convoked by the World Health Organization in Oslo, 1958 proposed a tentative typing of primary epithelial lung tumours.

The criteria have been tested at the International Reference Centre for Lung

Tumours for two years, and the present paper reports on the usefulness of the criteria, add a few details and discuss technical difficulties in interpretation and evaluation.

### REFERENCES

Kreyberg, L.—(1937) *Norsk Mag. Lægevidensk.*, **98**. Suppl. Forh. i Det Norske Medicinske Selskab, 36.
*Idem.*—(1954) *Brit. J. Cancer*, **8**, 199.
*Idem.*—(1956) *Brit. J. prev. soc. Med.*, **10**, 145.
*Idem.*—(1959) *Acta Un. int. Cancr.*, **15**, 78.
Nash, A. D. and Stout, A. P.—(1958) *Cancer*, **11**, 369.
World Health Organization.—(1960) *Epidemiology of Cancer of the Lung. Tech. Rep. Wld Hlth Org.*, No. 192, Genève.

### APPENDIX

### COMBINED STAINING OF KERATIN AND MUCUS

(Haemalum-Erythrosin-Saffron (Masson) and Alcian Green)

Bring sections to water.
Stain nuclei with Mayer's haemalum.
Blue in water.
Stain with erythrocin (1 per cent aqueous sol.), 3 min.
Rinse in water.
Differentiate quickly in 80 per cent alcohol.
Stain with Alcian Green, 5 min.
Rinse in water.
Cover sections with Saffron solution.
Drain, blot with filter paper.
Dehydrate in 3 changes of absolute alcohol.
Clear and mount in Canada balsam.

*Alcian Green :*

| | |
|---|---|
| Alcian Green (1 per cent aqueous solution) | 50 c.c. |
| Acetic acid (1 per cent) | 50 c.c. |
| Thymol | 20 mg. |

Filter before use.

*Saffron :*

| | |
|---|---|
| Saffron (dried stamens) | 6 g. |
| Distilled water | 300 c.c. |

Boil 1–2 hours in a water bath.
Cool, filter and add :

| | |
|---|---|
| Tannic acid (5 per cent) | 3 c.c. |
| Formol (40 per cent) | 3 c.c. |

(WHO International Centre for Lung Tumours, Rikshospitalet, Oslo.)


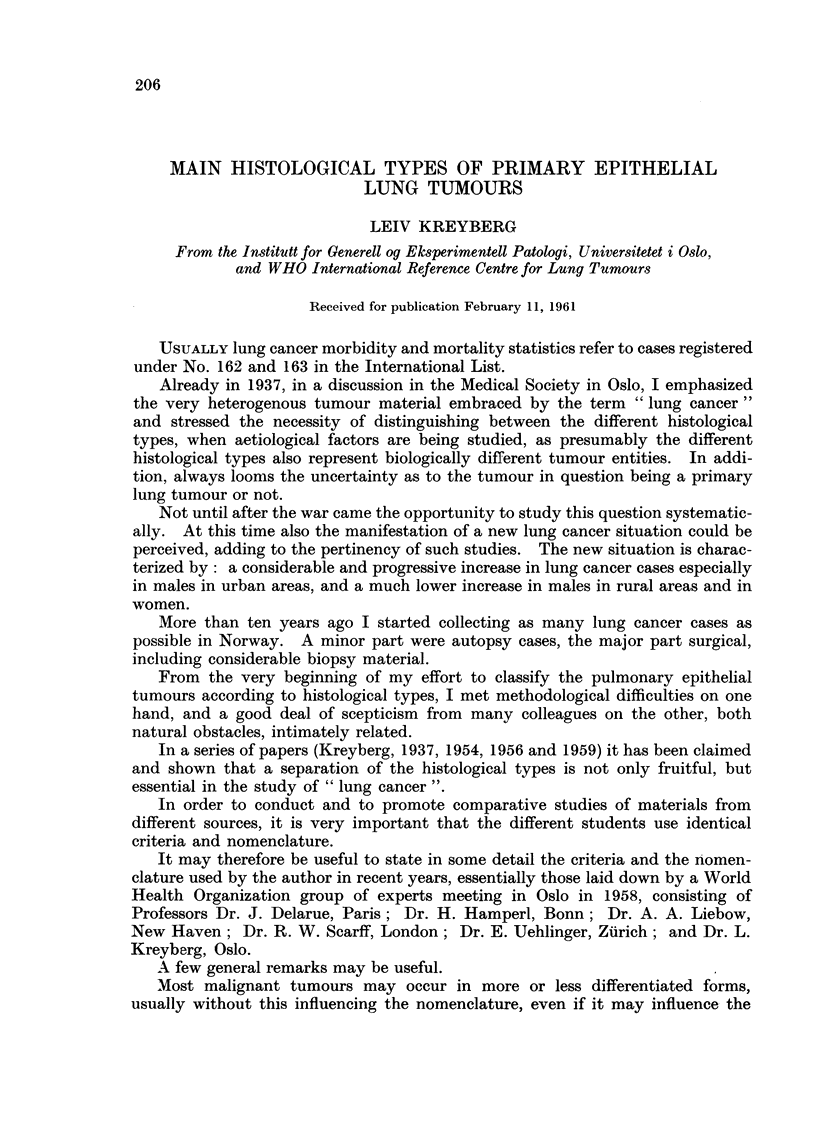

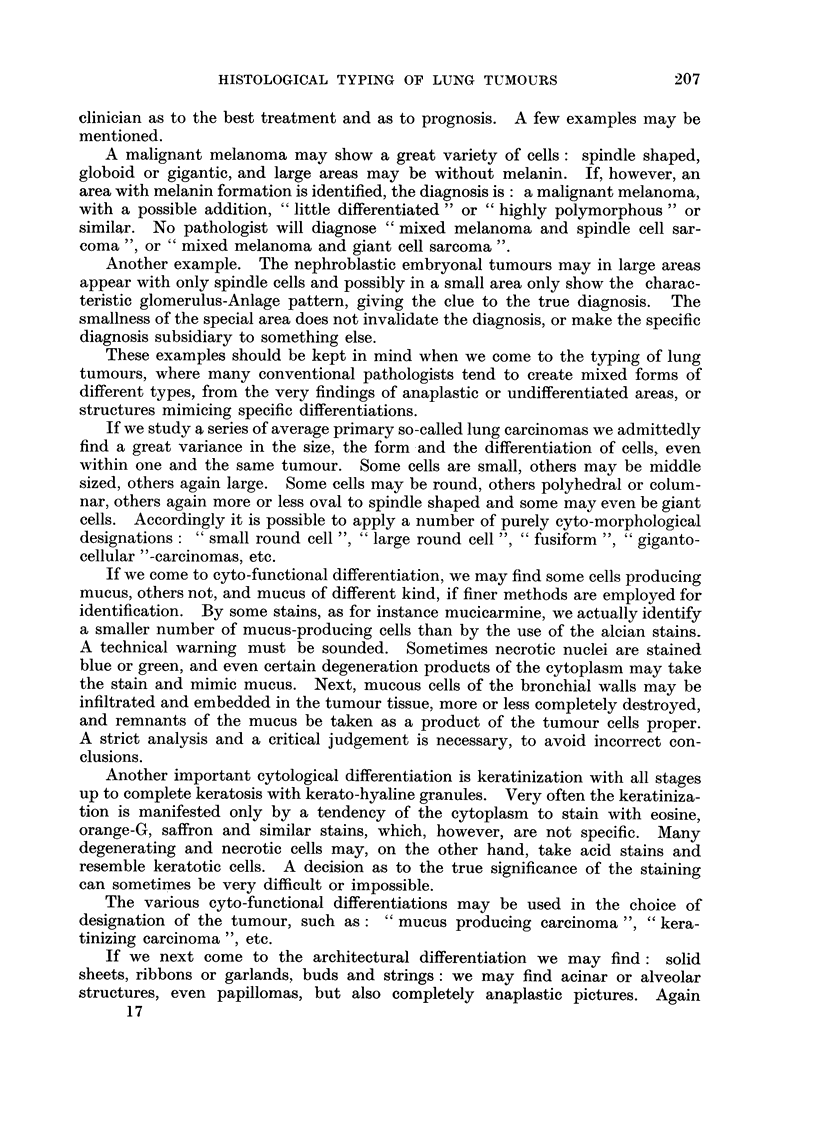

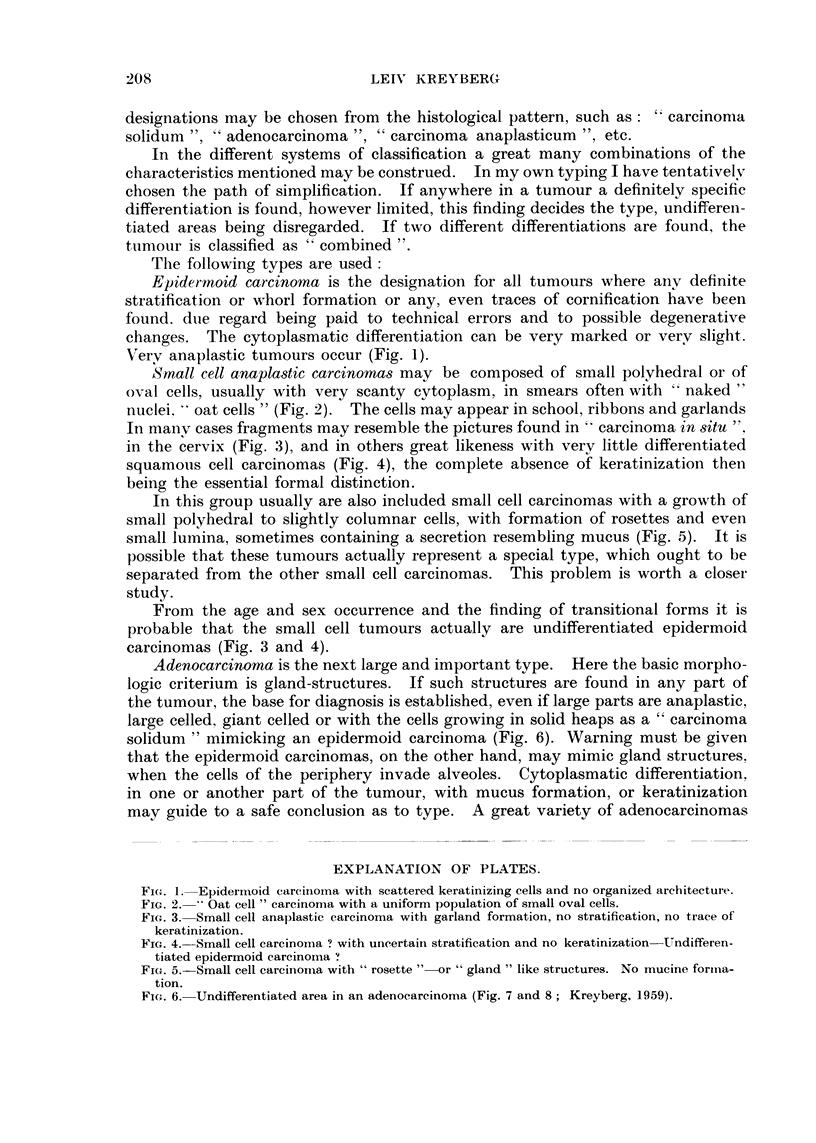

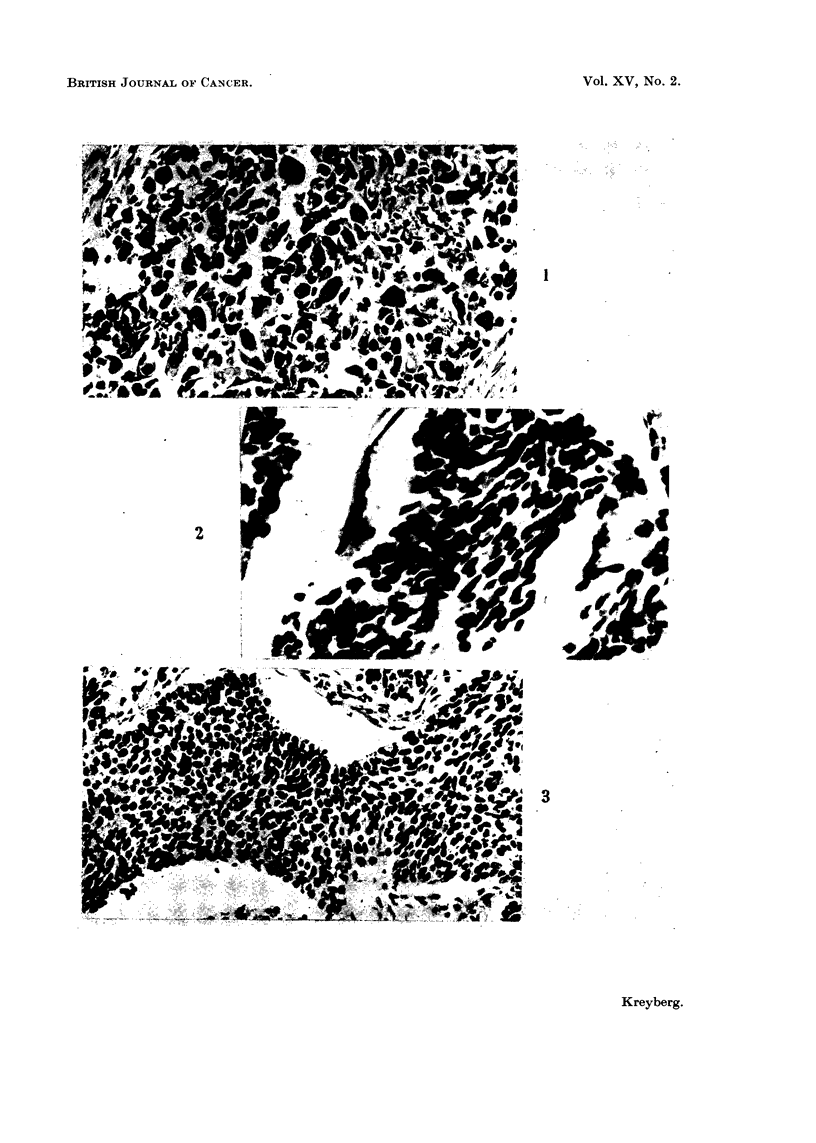

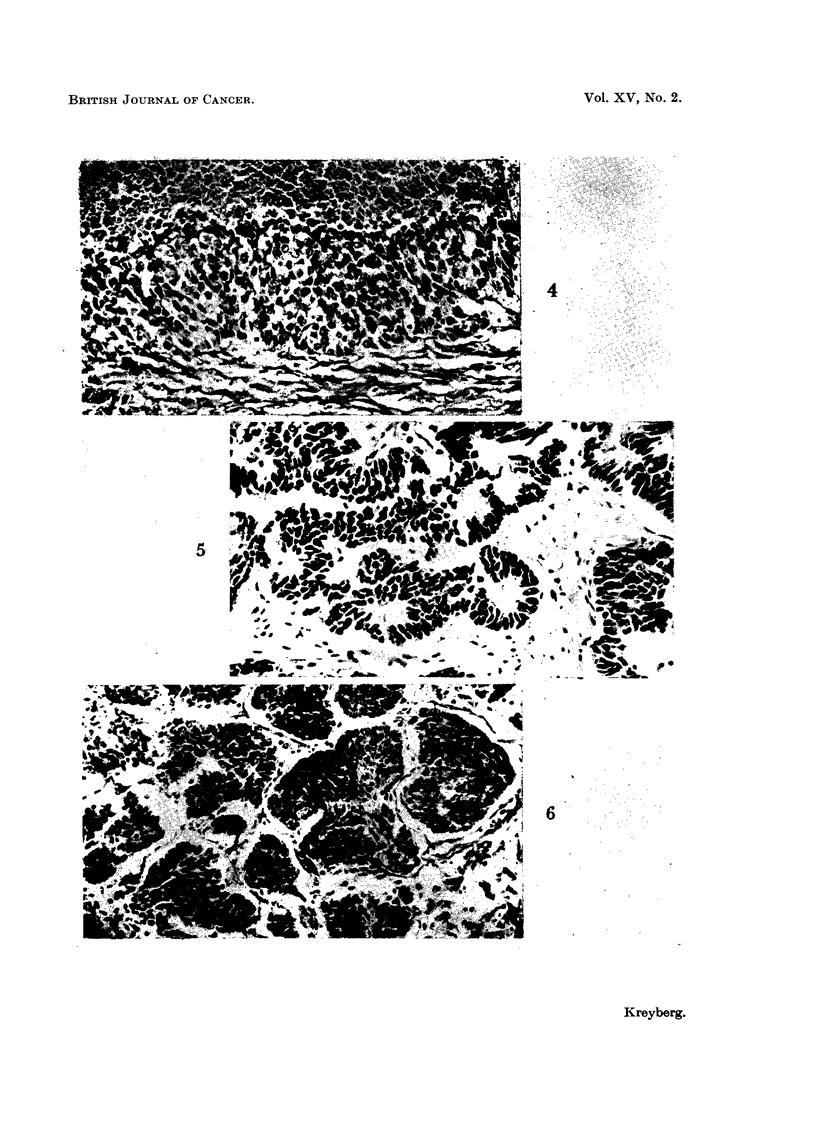

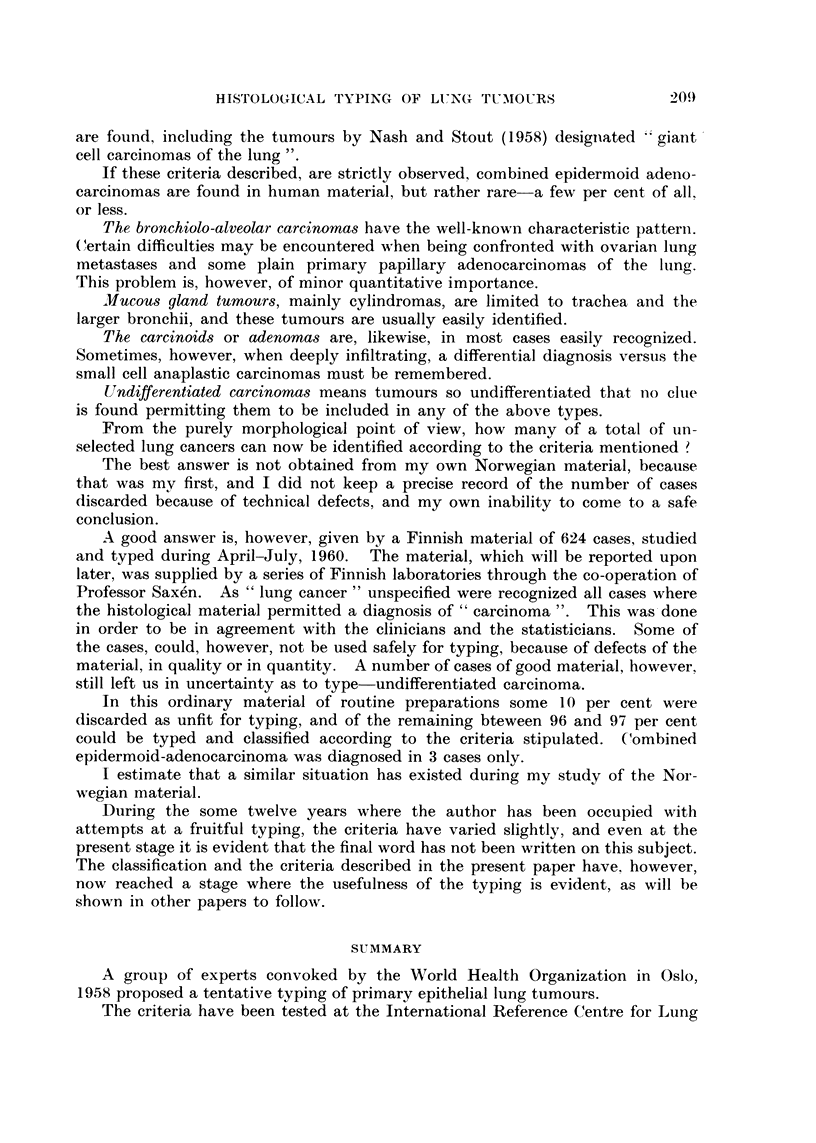

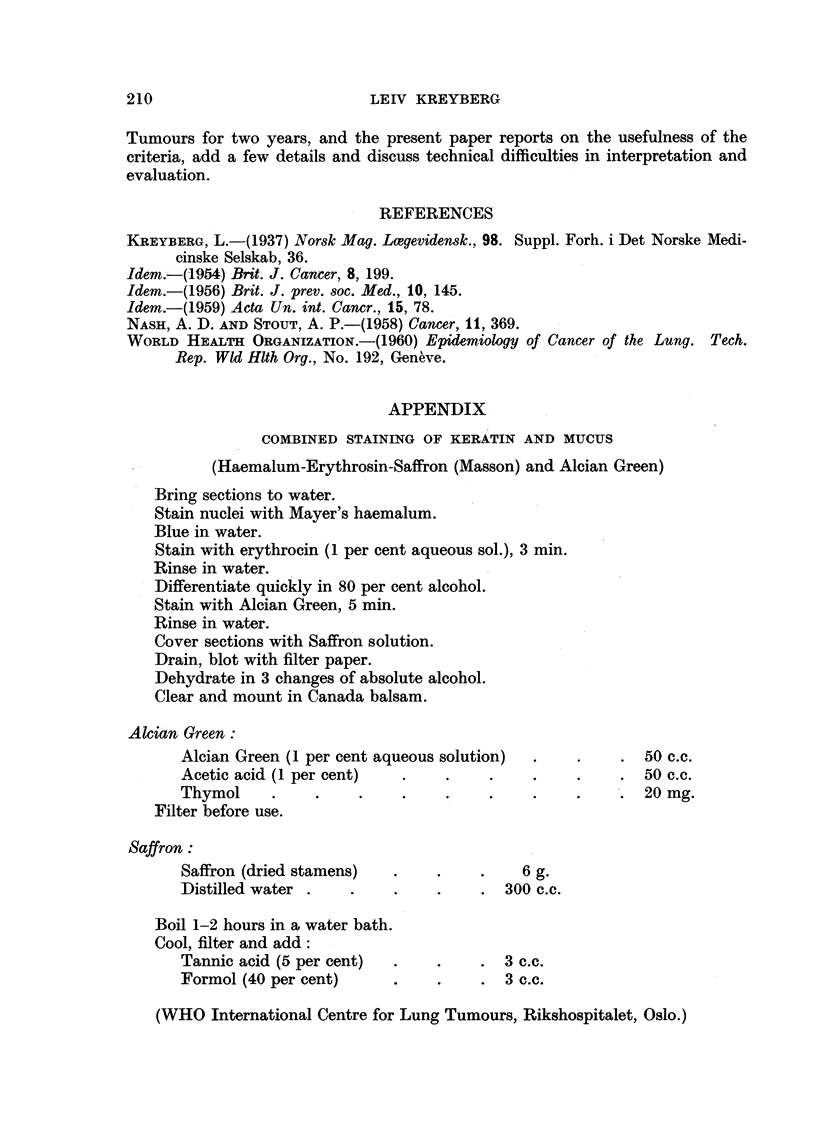


## References

[OCR_00321] NASH A. D., STOUT A. P. (1958). Giant cell carcinoma of the lung; report of 5 cases.. Cancer.

